# Intravenous immunoglobulin and intravenous methylprednisolone as optimal induction treatment in chronic inflammatory demyelinating polyradiculoneuropathy: protocol of an international, randomised, double-blind, placebo-controlled trial (OPTIC)

**DOI:** 10.1186/s13063-021-05083-1

**Published:** 2021-02-19

**Authors:** S. R. M. Bus, L. Zambreanu, A. Abbas, Y. A. Rajabally, R. D. M. Hadden, R. J. de Haan, C. A. J. M. de Borgie, M. P. Lunn, I. N. van Schaik, F. Eftimov, on behalf of the OPTIC study group, A. F. J. E. Vrancken, P. A. van Doorn, J. G. J. Hoeijmakers, N. Voermans, P. W. Wirtz, M. Padberg, M. de Rijk, G. Chavada, J. Miller, J. Holt

**Affiliations:** 1 Department of Neurology, Amsterdam Neuroscience, Amsterdam UMC, University of Amsterdam, Amsterdam, the Netherlands; 2 Department of Neurology, National Hospital for Neurology and Neurosurgery, Centre for Neuromuscular Disease, London, UK; 3 Department of Neurology, University Hospitals of Birmingham, Regional Neuromuscular Service, Birmingham, UK; 4 Department of Neurology, King’s College Hospital, London, UK; 5 Clinical Research Unit, Amsterdam UMC, University of Amsterdam, Amsterdam, the Netherlands; 6 Spaarne Gasthuis, Haarlem, the Netherlands

**Keywords:** Chronic inflammatory demyelinating polyradiculoneuropathy, CIDP, Randomised controlled trial, RCT, Corticosteroids, Methylprednisolone, IVMP, Intravenous immunoglobulins, IVIg

## Abstract

**Background:**

International guidelines recommend either intravenous immunoglobulin (IVIg) or corticosteroids as first-line treatment for chronic inflammatory demyelinating polyradiculoneuropathy (CIDP). IVIg treatment usually leads to rapid improvement and is generally safe, but does not seem to lead to long-term remissions. Corticosteroids act more slowly and are associated with more side effects, but may induce long-term remissions. The hypothesis of this study is that combined IVIg and corticosteroid induction treatment will lead to more frequent long-term remissions than IVIg treatment alone.

**Methods:**

An international, randomised, double-blind, placebo-controlled trial, in adults with ‘probable’ or ‘definite’ CIDP according to the EFNS/PNS 2010 criteria. Three groups of patients are included: (1) treatment naïve, (2) known CIDP patients with a relapse after > 1 year without treatment, and (3) patients with CIDP who improved within 3 months after a single course of IVIg, who subsequently deteriorate at any interval without having received additional treatment. Patients are randomised to receive 7 courses of IVIg and 1000 mg intravenous methylprednisolone (IVMP) (in sodium chloride 0.9%) or IVIg and placebo (sodium chloride 0.9%), every 3 weeks for 18 weeks. IVIg treatment consists of a loading dose of 2 g/kg (over 3–5 days) followed by 6 courses of IVIg 1/g/kg (over 1–2 days). The primary outcome is remission at 1 year, defined as improvement in disability from baseline, sustained between week 18 and week 52 without further treatment. Secondary outcomes include changes in disability, impairment, pain, fatigue, quality of life, care use and costs and (long-term) safety.

**Discussion:**

In case of superiority of the combined treatment, patients will experience the advantages of two proven efficacious treatments, namely rapid improvement due to IVIg and long-term remission due to corticosteroids. Long-term remission would reduce the need for maintenance IVIg treatment and may decrease health care costs. Additionally, we expect that the combined treatment leads to a higher proportion of patients with improvement as some patients who do not respond to IVIg will respond to corticosteroids. Risks of short and long-term additional adverse events of the combined treatment need to be assessed.

**Trial registration:**

ISRCTN registry ISRCTN15893334. Prospectively registered on 12 February 2018.

## Administrative information

**Table:** 

Title {1}	Intravenous immunoglobulin and intravenous methylprednisolone as optimal induction treatment in chronic inflammatory demyelinating polyradiculoneuropathy: protocol of an international, randomised, double-blind, placebo-controlled trial (OPTIC).
Trial registration {2a and 2b}.	ISRCTN registry (ISRCTN15893334), as of February 12th 2018
Protocol version {3}	V3.5.15MAY2020
Funding {4}	Princess Beatrix Spierfonds (Dutch Charity): grant ZonMW (Netherlands Organization for Health Research and Development for health related funding): grant GBS/CIDP Foundation International: grant GAIN UK: grant Sanquin Plasma Products (Dutch Blood Supply Foundation): logistical support
Author details {5a}	S.R.M. Bus^1^, L. Zambreanu^2^, A. Abbas^3^, Y.A. Rajabally^3^, R. Hadden^4^, R.J. de Haan^5^, C.A.J.M. de Borgie^5^, M.P. Lunn^2^, I.N. van Schaik^1, 6^, F. Eftimov^1^, on behalf of the OPTIC-study group. 1) Amsterdam UMC, University of Amsterdam, Department of Neurology, Amsterdam Neuroscience, Amsterdam,the Netherlands; 2) National Hospital for Neurology and Neurosurgery, Centre for Neuromuscular Disease, London, United Kingdom; 3) University Hospitals of Birmingham, Regional Neuromuscular Service, Birmingham, United Kingdom; 4) King’s College Hospital, Department of Neurology, London, United Kingdom; 5) Amsterdam UMC, University of Amsterdam, Clinical Research Unit, Amsterdam, the Netherlands; 6) Spaarne Gasthuis, Haarlem, the Netherlands.
Name and contact information for the trial sponsor {5b}	Sponsor: Prof. B.M.J. Uitdehaag, MD, PhD, neurologist, Head of Department. Amsterdam UMC, University of Amsterdam, Department of Neurology, Amsterdam Neuroscience, Amsterdam, the Netherlands. Telephone: (+ 31) 020 566 3942 Contact information coordinating principal investigator (PI): F. Eftimov, MD, PhD, neurologist. Amsterdam UMC, University of Amsterdam, Department of Neurology, Amsterdam Neuroscience, Amsterdam, the Netherlands. Room H2-213. Meibergdreef 9, 1105 AZ. Amsterdam, the Netherlands. Telephone: (+31) 020 566 9111, Email: f.eftimov@amsterdamumc.nl
Role of sponsor {5c}	The sponsor played no role in grant applications, the design, data collection, trial management, data analysis and interpretation. These tasks were the responsibility of the steering committee (see below under 5d). University College London Hospitals (UCLH) are the delegated coordinator in the United Kingdom (UK). This manuscript was written by the authors ({5a}). This manuscript is based on protocol version 3.5.15MAY2020. Decision to submit this manuscript was made by the steering committee. Trial results will be published in an international peer-reviewed journal. The steering committee and writing committee will have full access to data after completion of the trial and will make the final decision to submit the manuscript. Funders have no role in study design, data collection and analysis, decision to publish, or preparation of future manuscript(s).

## Introduction

### Background and rationale {6a}

Chronic inflammatory demyelinating polyradiculoneuropathy (CIDP) is an immune-mediated neuropathy affecting the arms and legs [1, 2]. Treatment of CIDP can be divided into (short-term) induction treatment and (long-term) maintenance treatment. First choice induction treatment recommended by the international European Federation of Neurological Societies/Peripheral Nerve Society (EFNS/PNS) guideline consists of either intravenous immunoglobulin (IVIg) or corticosteroids [3]. Several randomised controlled trials with different designs have demonstrated efficacy of these treatments [4, 5]. In responders, IVIg leads to improvement in most patients within 6 weeks of treatment [6]. Time to improvement with pulsed high-dose corticosteroids is longer, around 3 to 4 months [5]. The more rapid improvement with IVIg as well as the arguably preferential adverse event (AE) profile compared to corticosteroid treatment has made IVIg the first choice for most patients and physicians in the Netherlands, but also in other high-income countries [3, 7]. For the same reasons, IVIg is advocated for more severely affected patients and for long-term maintenance treatment. However, an important advantage of corticosteroids compared to IVIg is that they may lead to long-term remissions, defined as a stable condition without the need for continued treatment [8, 9]. In a recent open-label non-controlled pilot study, 20 CIDP patients were treated with a combination of IVIg and intravenous methylprednisolone (IVMP) over 4 months after which treatment was stopped. The majority of patients who completed the treatment protocol were in remission at 1 year. No patients discontinued treatment due to AEs associated with methylprednisolone [10]. We hypothesise that combining IVIg and pulsed IVMP as induction treatment will lead to more long-term remissions than IVIg monotherapy in patients with CIDP.

## Objectives {7}

The primary objective of this randomised controlled trial is to compare IVMP versus placebo in CIDP patients receiving IVIg and assess whether patients who receive IVMP achieve long-term remission more frequently than patients who receive placebo. The secondary objectives are to assess whether the combination of IVIg and IVMP, compared to IVIg alone, leads to:

- More frequent improvement
- More rapid improvement
- More or less adverse events
- Reduced healthcare costs

## Trial design {8}

The OPTIC trial is an international, multicentre, randomised, double-blind, placebo-controlled, parallel group superiority trial with two treatment arms allocated in a 1:1 ratio.

## Methods: participants, interventions and outcomes

### Study setting {9}

The trial will take place in five tertiary neuromuscular centres and three large general neurology departments in the Netherlands as well as six tertiary neuromuscular centres in the United Kingdom (UK). Participating investigators and centres are listed on page 35. The expected inclusion ratio between the Netherlands and the UK is 2:1.

### Eligibility criteria {10}

#### Inclusion criteria

We will include patients of 18 years of age or more with ‘probable’ or ‘definite’ CIDP according to the EFNS/PNS 2010 criteria [3], comprising three patient groups:

1. Treatment naïve
2. Previously treated patients who have a relapse* after a remission of at least 1 year
3. Patients treated with a single loading dose of IVIg in the last 3 months who achieved either a subjective or objective improvement and subsequently had a deterioration as judged by his or her treating physician (for definition of deterioration see paragraph 4.1).

* A relapse or deterioration after treatment is defined as any deterioration warranting treatment as judged by the treating physician.

#### Exclusion criteria

1. Presence of IgM paraproteinemia and/or anti-MAG antibodies or known CIDP-specific antibodies associated with poor treatment response to IVIg
2. Use of drugs known to cause a demyelinating neuropathy
3. Use of any immunosuppressive or immunomodulatory drugs in previous 6 months (except for a single loading dose of IVIg within 3 months or low dose prednisolone (20 mg or less) for a short period not exceeding 2 weeks)
4. Known serious adverse events (SAEs) with previous IVIg or corticosteroid treatment. Hypersensitivity to methylprednisolone or any component of the formulation. Hypersensitivity to the human immunoglobulins or to any of the excipients. Selective IgA deficiency patients who developed antibodies to IgA
5. Systemic fungal infections, unless specific anti-infective therapy is employed
6. Known hyperprolinaemia type I or II or known fructose intolerance
7. One of more of the risk factors associated with increased risk of AEs of IVIg or IVMP or conditions that could lead to unblinding of treatment (i.e. diabetes, IgA deficiency, gastric ulcers, psychosis, severe hypertension (180/110 mmHg or more on repeated measurements), hypocalcaemia (lower than 2.20 mmol/L, corrected for albumin), moderate or severe heart failure, severe cardiovascular disease (i.e. more than one myocardial infarction and or ischemic stroke), renal failure (glomerular filtration rate < 30 ml/min))
8. History of osteoporosis or osteoporotic fractures
9. Known active malignancy, currently treated with chemotherapy or immunomodulatory drugs, or with a life expectancy of less than 1 year
10. Bodyweight more than 120 kg
11. Pregnancy or nursing mother; intention to become pregnant during the course of the study; female patients of childbearing potential either not using or not willing to use a medically reliable method of contraception for the entire duration of the study. A woman is considered of childbearing potential from menarche and until becoming post-menopausal, unless permanently sterile. Permanent sterilisation methods include hysterectomy, bilateral salpingectomy and bilateral oophorectomy. A postmenopausal state is defined as no menses for 12 months without an alternative medical cause. Acceptable methods of contraception are combined (oestrogen and progestogen containing) hormonal contraception associated with inhibition of ovulation (whether oral, intravaginal or transdermal), progestogen-only hormonal contraception associated with inhibition of ovulation (whether oral, injectable or implantable), progestogen-only oral hormonal contraception, where inhibition of ovulation is not the primary mode of action, male or female condom with or without spermicide, cap or diaphragm, intrauterine device (IUD), intrauterine hormone-releasing system (IUS), bilateral tubal occlusion and vasectomised partner (provided that partner is the sole sexual partner of the trial participant and that the vasectomised partner has received medical assessment of the surgical success)
12. Known cataract or cataract obvious on fundoscopy
13. Current psychosis or past history of psychosis
14. Poor dental status
15. Known pulmonary embolism or other deep venous thrombosis in patient’s medical history, without current anticoagulant therapy
16. Adults lacking capacity to give informed consent (IC)
17. Lack of written IC

### Who will take informed consent? {26a}

The treating neurologist or an authorised delegate, such as a trained research nurse, obtains IC. After an explanation by the PI or an authorised surrogate and after reading the patient information sheet, patients are asked to consider participation. If necessary, further explanation will be provided.

### Additional consent provisions for collection and use of participant data and biological specimens {26b}

Additional consent is requested to allow for participants to be notified of possible follow-up studies, request relevant contact information and be informed which study treatment they received during the trial. This trial does not involve collecting biological specimens.

### Interventions

**Fig. 1:**
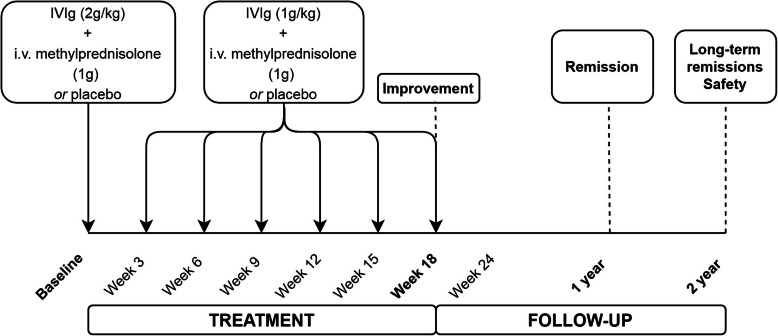
Treatment protocol and follow-up

Patients will be randomised to receive 7 infusions of 1000 mg IVMP (intervention) or placebo (comparator) in combination with IVIg (standard care), every 3 weeks during the 18-week intervention period (Fig. 1; see the ‘Participant timeline {13}’ section). Both study arms receive IVIg, which consists of a loading dose (2 g/kg divided over 2–5 days) and 6 maintenance treatments of 1 g/kg (given over 1–2 days), as well as weekly alendronic acid 70 mg and daily calcium/vitamin D 500 mg/800 IU (co-medication) to reduce the chance of corticosteroid-induced AEs according to national guidelines [11]. All brands of IVIg are allowed.

#### Explanation for the choice of comparators {6b}

The comparator consists of sodium chloride 0.9% (placebo).

##### Dosage and administration

The dose of sodium chloride 0.9% is 100 ml and will be administered by intravenous infusion. Sodium chloride 0.9% was chosen as comparator treatment as it appears identical to the intervention treatment, which is prepared using a sodium chloride solution. Sodium chloride is widely used as a solvent and it has few side effects. Large amounts of sodium chloride can cause peripheral oedema, hypernatremia and thrombophlebitis. These adverse events are not expected given the small amounts of sodium chloride used in this study (100 ml every 3 weeks). Preparation, labelling and administration are covered under the ‘Intervention description {11a}’ section.

#### Intervention description {11a}

Interventional treatment consists of intravenous methylprednisolone.

##### Dosage and administration

The IVMP dose is 1000 mg in 100 ml sodium chloride 0.9% solution. Venous access and administration of treatment will be provided by hospital nurses or by Sanquin Home Care services. In the Netherlands, the first treatment will be administered in a hospital or day care centre and the remaining infusions in a hospital, day care centre or at home (Sanquin Thuisservice, NL) according to local treatment protocols. In the UK, all treatments will be administered in a hospital. IVMP (or placebo) will be administered during 60 min, prior or after IVIg infusion, depending on logistical preference of participating hospitals and home care services. IVIg and IVMP/placebo may be administered on the same day, but not simultaneously. For each treatment interval, a window of 2 days is accepted for administration of study treatment.

##### Preparation of IVMP and placebo

In the Netherlands, IVMP vials will be provided with a Good Manufacturing Practice (GMP) annex 13 label by the trial pharmacy of the Amsterdam UMC, location AMC (referred to as ‘AMC trial pharmacy’). AMC trial pharmacy will distribute the vials to the pharmacies of the participating hospitals in the Netherlands. Local pharmacies are allowed to order the methylprednisolone, after approval by the Medical Research Ethics Committee (MREC). Pharmacies are allowed to use their sodium chloride 0.9% from stock to prepare the placebo. For administration in the participating centres, IVMP/placebo (or: investigational medical product, IMP) will be prepared and labelled locally. For treatments at home (only in the Netherlands), IMP will be prepared by the AMC trial pharmacy. IMP will be labelled according to GMP (annex 13), in line with the pharmacy manual provided by the AMC trial pharmacy. AMC trial pharmacy and pharmacies of the participating centres will be aware of patient treatment allocation and will dispense accordingly. A second pharmacist will control dispensing of correct dose and treatment allocation. In the UK, pharmacies may use local stock for IMP. Preparation of IMP will be performed by the hospital pharmacist or an (unblinded) nurse, depending on local pharmacy protocols. When IMP preparation and labelling are performed by an unblinded nurse on the ward, a different, blinded nurse will subsequently administer treatment. Study medication, including placebo, will be put into indistinguishable infusion sacks to ensure adequate blinding. Sacks will be labelled according to institutional standard operation procedures (SOP’s) and national guidelines and regulation. At least the following items are present on the label: name of the patient, name of the study, the content (noted as follows to include both IMPs: ‘methylprednisolone 1 gram *or* placebo’, to prevent unblinding) and expiration date. After preparation, study drug and infusion material will be distributed to the departments of the participating centres or to patient’s home address where home-care nurses administer treatment. In the Netherlands, Sanquin Blood Supply will distribute IMPs for home care. AMC trial pharmacy supplies the medication to Mediq pharmacy. For transportation of IMP to patient’s home, infrastructure of Mediq pharmacy in place for IVIg distribution will be used, in line with Good Distribution Practice (GDP). Administration of IVMP has to start within 7 days after preparation (transfer of IVMP solution from to infusion bags/containers) in cooled condition, per summary of product characteristics (SmPC) of IVMP.

#### Criteria for discontinuing or modifying allocated interventions {11b}

Changes in IMP dosing due to potential AEs will not be allowed. Decision to postpone or not administer a single IMP infusion (for example in case of adverse events or new contra-indication to administer IMP) or to discontinue further IMP treatment altogether will be made by the treating physician, if necessary, after deliberation with PI. Any changes in IMP regimen will be discussed with the coordinating PI prior to implementation and recorded as a protocol deviation.

#### Strategies to improve adherence to interventions {11c}

Adherence to intervention protocol is maximised by careful counselling of participants and training of study staff (including home care nurses) and monitored using drug accountability logs. If necessary, training is repeated during the course of the study. All nurses involved in IMP preparation and administration in the UK will undergo Good Clinical Practice (GCP) training and protocol training. Nurses will record the administration date and time of infusion (start to end), infusion speed, blood pressure (before and after) and AEs in a logbook. Blood glucose measurements will not be taken during or directly after an infusion, even if required in local protocols. Logbooks of administration at participating centres will be kept by the local investigator. At the end of individual treatment period, study data will be added to the electronic case report forms (eCRF). Logbooks from the pharmacists, local investigators, and Sanquin Home Care services will be compared with the dispensing sheets of the trial pharmacist after trial has ended and blind has been broken. In the UK, two separate logbooks will be used, one to be completed by the unblinded nurse preparing the drug, and one to be completed by the blinded nurse administering the infusion of IMP.

#### Relevant concomitant care permitted or prohibited during the trial {11d}

The decision to change IVIg treatment regimen (i.e. following adverse events or new contra-indication) or to discontinue IVIg treatment altogether will be made by the treating physician, if necessary, after discussion with PI. Changes will be recorded as a protocol deviation. IMP administration will continue according to protocol regardless of changes in IVIg treatment. Use of any immunosuppressive co-medication 6 months prior to the study is an exclusion criterion. Anti-histamines are frequently used to prevent or reduce skin reactions following IVIg treatment and are allowed during the study. Proton-pump inhibitors (PPI) or H2 antagonists can be used to treat any dyspepsia caused by either steroids, or IVIg, or alendronate. Changes in other co-medication during the study are allowed, as one does not expect this to influence the disease course. Administration of live or live attenuated vaccines is contraindicated in patients receiving immunosuppressive doses of corticosteroids; however, any vaccination is allowed up to 6 weeks before the first study treatment and 6 weeks after the last study treatment.

### Provisions for post-trial care {30}

After study completion, patients will continue to receive standard care for CIDP. Patient who are stable will not require additional treatment. Patients who reach a preliminary endpoint, defined as deterioration during the 18-week treatment period requiring additional CIDP treatment, no improvement at week 18, restart CIDP treatment for any reason (i.e. a relapse) between week 18–52 or drop out during study for whatever reason will be treated at physician’s discretion and will complete follow-up visits according to protocol. AMC Medical Research BV (AMR) has an insurance which is in accordance with the legal requirements in the Netherlands (Article 7 of the Medical Research Involving Human Subjects Act (WMO) and the Measure regarding Compulsory Insurance for Clinical Research in Humans of 23 June 2003). This insurance provides cover for damage to research patients through injury or death caused by the study for patients in the Netherlands. Where harm to participants arises from the conduct of the research, National Health Services (NHS) indemnity applies. Where harm to participants comes from the design or management of the research, this comes under Sponsor insurance. These arrangements are covered in a coordinator agreement and in the site agreements.

### Outcomes {12}

#### Primary outcome

The primary outcome is the number of patients in remission at 52 weeks after start of treatment. Remission is defined as sustained improvement without the need for further treatment. Improvement is defined as improvement by at least the minimal clinical important difference (MCID) on the inflammatory Rasch-Overall Disability Scale (I-RODS) and/or improvement of one or more points on the adjusted Inflammatory Neuropathy Cause and Treatment Disability Scale (INCAT-DS) at 18 weeks (end of treatment) compared to baseline. For the I-RODS, MCID is calculated using a statistical approach in which an increase of 1.96 SE is considered relevant improvement [12]. For the adjusted INCAT-DS, a decrease of 1 point or more is considered a relevant improvement [13, 14]. Changes in upper limb function from ‘0’ (normal) to ‘1’ (minor symptoms) or from ‘1’ to ‘0’ are not considered relevant deterioration or improvement [13]. Sustained is defined as no deterioration between 18 and 52 weeks, i.e. difference on the I-RODS of less than the MCID difference and/or less than one point on the adjusted INCAT-DS. Patients will be considered a treatment failure if they (1) received additional CIDP treatment during the 18-week intervention period, (2) do not improve at 18 weeks, (3) restart CIDP treatment for any reason between 18 and 52 weeks, or (4) do not show a sustained improvement at 52 weeks as defined above.

#### Secondary outcomes

Secondary outcomes will be assessed at 18 weeks, 52 weeks and 104 weeks, or earlier if a preliminary endpoint is reached. Secondary outcomes include:

1. The number of patients with improvement on disability equal or more than the MCID
2. Time to MCID improvement on disability
3. Mean change in disability
4. Mean change in grip strength
5. Mean change in muscle strength
6. Mean change in sensory impairment
7. Mean change in fatigue
8. Mean change in pain
9. Mean change in health-related quality of life (HRQL)
10. Care use and overall healthcare-related costs
11. Number of (S) AEs (including corticosteroid associated AEs), as defined in the ‘Adverse event reporting and harms {22}’ section

#### Outcome measures

For disability, we will use the I-RODS and the INCAT-DS. The I-RODS is a patient self-report linearly weighted scale that measures activity and social participation limitations [12, 15]. The I-RODS consists of 24 questions and allows for three response options: (0) impossible to perform, (1) performed with difficulty and (2) easily performed. Scores range from ‘0’ (all activities impossible to perform) to ‘48’ (all activities easily performed). These scores are transformed and expressed in log-odds (logits), ranging from − 6.95 to 8.11. The I-RODS has been developed specifically for inflammatory neuropathies including CIDP and has been validated in a large group of patients [16]. In addition, disability will be measured using the adjusted INCAT-DS, which has been used in numerous CIDP trials [5, 13, 17]. The adjusted INCAT-DS is a 10-point ordinal scale measuring limb disability, ranging from ‘0’ (no arm or leg disability) to ‘10’ (no arm movement possible, wheelchair bound). The reason to choose both disability scales to assess improvement and remission is outlined in the discussion. We will use a handheld Vigorimeter to measure grip strength in kPa, the Medical Research Council (MRC) sum score of 12 predefined muscle groups (including shoulder abduction, elbow flexion, wrist extension, hip flexion, knee extension and foot dorsiflexion) to measure muscle strength, the INCAT- sensory sum score (INCAT-SS) to assess sensory impairment, the Rasch-built Fatigue Severity Scale (R-FSS), the Pain intensity numeric rating scale (PI-NRS) and the EuroQol (EQ-5D-5L) to measure HRQL [18–24]. Health care resources will be measured using working documents and the iMTA Medical Consumption Questionnaire (*i*MCQ) and the Productivity Cost Questionnaire (*i*PCQ), both adapted to the study population [25, 26]. Working documents keep track of IVIg use, outpatient hospital consultations, hospitalizations, and major diagnostic examinations and therapeutic interventions. Tailored *i*MCQ will be used to measure volume of out-of-hospital consultations (general physician, physical therapist, psychologist, etc.), other institutionalizations (temporary rehabilitation, nursing home), day-care treatment, emergency admissions and out-of-pocket expenses by patients. The impact of CIDP on the ability of a person to perform work, production loss following absence from work or personal inefficiencies will be measured with the *i*PCQ. AEs reported spontaneously at visits or otherwise reported by patient or observed by physician, during the first 24 weeks of the study, or in the case of an early endpoint during treatment protocol within 6 weeks of last treatment course, are recorded using an AE template provided by the Amsterdam UMC, Clinical Research Unit (CRU). The AE template consist of a description, severity score (mild, moderate, severe), potential relation to IMP (unlikely, unlikely, possible, probable, definite, not possible to assess), consequences for IMP administration (options including temporary or permanent stops, dose changes), any other actions taken (i.e. start of co-medication, a referral) and the outcome (i.e. resolved). Severity scores will be defined as followed: mild (i.e. an AE that is transient and may require only minimal treatment or therapeutic intervention; the event does not generally interfere with usual activities of daily living), moderate (i.e. alleviated with specific therapeutic intervention; the event interferes with usual activities of daily living, causing discomfort but poses no significant or permanent risk of harm to the research patient) or severe (i.e. an AE that interrupts usual activities of daily living, or significantly affects clinical status, or may require intensive therapeutic intervention). Patients will complete a structured questionnaire with most frequent corticosteroid and IVIg-related AEs at the 18-week visit. These AEs are subsequently scored by the investigator using the previously described severity score. (S)AEs are further described under the ‘Adverse event reporting and harms {22}’ section. Long-term corticosteroid related AEs are also assessed by the investigator at weeks 52 and 104. AE questionnaires are completed after all other outcome assessments have been performed. Patients are asked to indicate which treatment they think they have received, at the end of the study. Outcome measures and assessment visits are listed under the ‘Plans for assessment and collection of outcomes {18a}’ section and shown in Table 1.

**Table 1:** Study visits and measurements

	Study visits							
	Intervention period (in weeks)				Follow-up period (in weeks)		Safety follow-up (in weeks)	Unscheduled visits (in weeks)
Measurements	BL	6	12	18	24	52	104	0–52
**Blood pressure**	X	X	X	X	X	X		X*
**Body weight**	X			X		X		X*
**HbA1C**	X				X	X		
**AEs**	X	X	X	X	X			X^‡^
**AE questionnaire**^**§**^				X		X	X	X^*¥^
**I-RODS**	X	X	X	X	X	X	X	X
**INCAT-DS**	X	X	X	X	X	X	X	X
**Grip strength**	X	X	X	X	X	X		X
**MRC sum score**	X	X	X	X	X	X		X
**INCAT-SS**	X	X		X		X		X*
**EuroQol**	X	X		X		X		X*
***i*MCQ**		X		X		X		X*
***i*PCQ**		X		X		X		X*
**R-FSS**	X	X		X		X		X*
**PI-NRS**	X	X		X		X		X*

*Abbreviations: BL* baseline, *AE* adverse event, *I-RODS* inflammatory Rasch Disability Scale, *MRC* Medical Research Council, *INCAT-DS* Inflammatory Neuropathy Cause and Treatment Disability Scale, *INCAT-SS* Inflammatory Neuropathy Cause and Treatment sensory sum score, *iMCQ* iMTA Medical Consumption Questionnaire, *iPCQ* iMTA Productivity Cost Questionnaire, *R-FSS* Rasch-built Fatigue Severity Scale, *PI-NRS* pain intensity numeric rating scale. *If the unscheduled visit is considered as a preliminary endpoint visit. ^**§**^This row includes the investigator assessed (long-term) AEs investigated at weeks 52 and 104. ^‡^If unscheduled visits takes place during treatment protocol, within 6 weeks of ending treatment protocol. ^¥^In case of a preliminary endpoint during intervention period, week 18 AE questionnaire is completed by patient and physician

### Participant timeline {13}

Total follow-up is 104 weeks (2 years). After the intervention period of 18 weeks, patients will be followed for another 86 weeks during which treatment will be given only in case of deterioration by at least the MCID on the I-RODS and/or deterioration (i.e. increase) of at least one point on the adjusted INCAT-DS. Choice of treatment in case of relapse is at discretion of the treating physician. If a change of treatment regimen is regarded necessary during the 18 weeks’ intervention period, trial medication will be stopped but further follow-up will continue as scheduled. Additional (ad hoc) visits, i.e. if a relapse is suspected, can be scheduled at discretion of treating physician. Treatment protocol and follow-up are illustrated in Fig. 1.

### Sample size {14}

There are no robust figures on the long-term remission rates in patients treated with IVIg only. In a previous trial, remission during a 6-month period was seen in about half of IVIg responders, compared to 100% in IVMP responders [9]. However, a significant proportion of patients were not treatment naïve but known responders to IVIg. Furthermore, follow-up was shorter than the follow-up period in this study. We assume that the remission rate after IVIg is approximately 35% during an 8-month follow-up period. Treatment-naïve CIDP patients treated with dexamethasone had a long-term remission rate of 56% using a more stringent definition of improvement compared to this study [5, 8]. Our limited prospective pilot data of the OPTIC protocol suggest similar remission rates (59%) [10]. We assume remission rates of 35% in the IVIg/placebo group, and 65% in the IVIg/IVMP. A 30% difference in remission rate is large but warranted as IVMP may lead to various AEs in patients and because there is the alternative of continuing maintenance IVIg treatment. A two-group chi-square test with a 0.05 two-sided significance level will have 80% power to detect the difference between a control group proportion (IVIg/placebo) of 0.35 and a treatment group proportion (IVIg/IVMP) of 0.65 (odds ratio of 3.45) when the sample size in each group is 43 (86 patients in total). Anticipating on a 10% attrition rate due to AEs, we will include 48 (43/0.90) patients in each group (96 patients in total).

### Recruitment {15}

Participants are enrolled in both tertiary referral centres as well as large general neurology clinics, with an adequate geographical spread in both the Netherlands and the UK. Design of the trial, with IVMP as an addition to standard care IVIg, and limited clinic visits over the 104-week course of the trial promote trial enrolment. Frequent trial updates in the form of newsletters and presentations at (inter) national (neuromuscular) conferences as well as patient events promote trial visibility amongst referring neurologists and participants.

### Assignment of interventions: allocation

#### Sequence generation {16a}

After signing the IC form, patients are randomised to either IVIg + IVMP or IVIg + placebo. Randomisation is performed using the functionality of Castor EDC. Randomisation is stratified per country (NL/UK) and previously administered treatment (treatment naïve versus previously treatment, inclusion group 2) with random permuted blocks of sizes 2 or 4 within the strata.

#### Concealment mechanism {16b}

Investigators and other study staff are unable to view treatment allocation in Castor EDC. Only the pharmacists are able to view treatment allocation in Castor EDC. A specifically designated co-investigator is the only person able to change user rights in the Castor EDC database. The database software (audit trail) automatically logs these changes. Randomisation code is provided to the pharmacist of the participating centre automatically through Castor following randomisation.

#### Implementation {16c}

Treating neurologists or assigned (GCP trained) delegates are allowed to enrol participants. To randomise a patient, local investigator provides patient’s personal identification number (PIN) and treatment status (previously treated versus treatment naïve) to the coordinating investigator (or assigned delegate) who will perform randomisation.

### Assignment of interventions: blinding

#### Who will be blinded {17a}

Patients, relatives, IMP administering nurses (in hospitals, day care centres, home-care), treating and evaluating physicians will be blinded for treatment allocation. The blind will be maintained until the whole study is completed.

#### Procedure for unblinding if needed {17b}

Pharmacists will be the only persons able to break the randomisation code. The randomisation code will be broken after the last follow-up visit of the last patient, after data cleaning and database lock. The randomisation code can be broken during the first 18 weeks if unblinding is judged necessary by treating physician to start additional treatment or to change the treatment regimen. These patients will be considered treatment failures and will reach a preliminary endpoint. All secondary outcome measurements will be performed before the blind is broken. In these cases, the coordinating PI will remain blinded. Treatment allocation will not be revealed in patients who are considered a treatment failure, but do not need or do not wish to receive additional treatment.

The randomisation code can be broken if a relation between trial treatment and a SAE is suspected and if knowledge of treatment allocation is deemed necessary by local investigator or treating physician. In case of a Suspected Unexpected Serious Adverse Reaction (SUSAR), the investigator will send an Unblinding Request Form (URF) to the coordinating PI and make every effort to contact the coordinating PI to discuss options. If unblinding is deemed necessary, coordinating PI will send the URF to the study pharmacist. The study pharmacist will reveal treatment allocation of the individual patient to an independent physician on the unblinding form (UF) to ensure continued blinding of local investigator and treating physician. The UF must include the date, time and reason for unblinding. This information will be recorded in the eCRF and local source documents. If the blind is broken, the patient must be discontinued from study medication as soon as possible. The patient will be encouraged to perform an end of study assessment and be under medical supervision until symptoms cease or the condition becomes stable. An independent physician will document the SUSAR in the appropriate Dutch module (ToetsingsOnline) in order to maintain the blind for other research team members.

##### Emergency unblinding procedure

There are no likely situations that would mandate emergency unblinding. Complications of corticosteroids are very well known and would not warrant change in management if treatment allocation was known, even in an emergency care setting. The comparator (placebo, normal saline) does not contain an active substance and would not produce any significant AEs. Clinical deterioration in CIDP, possibly requiring unblinding, is slowly progressive and its management is not expected to take place in an emergency care setting. Management of clinical deterioration due to a concomitant condition (i.e. infection) is not changed by knowledge of treatment allocation. In the UK, in the event that a patient, or their treating physician, feels there is a an urgent need to find out whether the patient is receiving steroids or placebo, they should contact the Neurology Registrar on call at the respective participating institution to explain why treatment allocation needs to be revealed as a matter of urgency. Each patient will carry a card containing brief information about the trial, in order to allow any Neurology Registrar to understand what the query refers to. If the Neurology Registrar on call agrees there is a potential need to reveal treatment allocation, they will contact the centre PI. Emergency unblinding is the sole responsibility of the investigator. In order to ensure that this can be achieved without significant delay, UK centre PI’s can nominate sub-investigators via the delegation log, who can authorise emergency unblinding in the event that the PI cannot be reached within 30 min. If the centre PI authorises unblinding, there will be two different procedures to follow: one for UCLH and one for the other five UK participating sites. If the local PI authorises emergency unblinding, the Neurology Registrars on call at King’s College Hospitals London, The Walton Centre Liverpool, University Hospitals of Birmingham, Royal Victoria Infirmary Newcastle, or Queen Elizabeth University Hospital Glasgow will contact the pharmacy who will perform the unblinding by accessing accountability records. At UCLH, the clinical trials pharmacist will, upon receiving treatment allocation, print this information and put it in a sealed envelope with a signature and a patient label across the seal. These envelopes will be stored in a locked drawer in the office of the local PI. In the event that emergency unblinding is authorised at UCLH, the Neurology Registrar on call will contact the security officer who will assist the Neurology on call doctor in accessing the PI’s office for access to the relevant letter.

### Data collection and management

#### Plans for assessment and collection of outcomes {18a}

Outcomes will be assessed by the local investigator, or delegated (and trained) person, at follow-up visits as listed in Table 1. Table 2 lists the consultation by telephone. Questionnaires are sent to patients before telephone consultations take place. A time window of 3 days is accepted for all study procedures. A time window of 2 and 4 weeks is allowed for the week 52 and week 104 visits, respectively. HbA1c is assessed as part of standard care at baseline, 24 weeks and 52 weeks. If HbA1c has been assessed in the work up leading to the diagnosis, up to 3 months prior to enrolment, this measurement is allowed as baseline value.

**Table 2:** Consultation by phone

	Consultation by phone (in weeks)
	3
**I-RODS**	X
**INCAT-DS**	X
**AEs**	X

*Abbreviations: I-RODS* inflammatory Rasch Disability Scale, *INCAT-DS* Inflammatory Neuropathy Cause and Treatment Disability Scale, *AE* adverse event

#### Plans to promote participant retention and complete follow-up {18b}

Patients who reach a preliminary endpoint or drop out of the study for whatever reason will be treated at physician’s discretion and will complete follow-up visits according to protocol. If patients are unable to complete follow-up, data obtained up until that point will be stored in eCRF and used in the analysis if appropriate. Additional visits are considered standard care and can be scheduled at the discretion of treating physician. There will be no replacement of individual patients after withdrawal.

#### Data management {19}

Source documents for each patient will be kept at the patient’s study site until the last follow-up visit of the last patient included. Records will include date of informed consent, visit dates, medical history, neurological examinations and study parameters, administration of other medication and (S)AEs. Data are recorded on a working document after which treating physician or research nurse will enter data in the eCRF. Patient questionnaires are completed on paper or electronically (and uploaded directly in database). After the end of study, individual patient data (pseudonymised source documents) are transferred and stored at the Trial office of the Department of Neurology, Amsterdam UMC, location AMC, Amsterdam, the Netherlands. After the end of the study, all essential forms pertaining to the conduct of the study will be archived by the investigator for a period of 15 years in accordance with the SOP of the Amsterdam UMC, location AMC. The coordinating PI and study sponsor are the only ones who have access to this information. All patient data are stored in a way that the privacy of participants is respected. Data validation procedures (such as reason for change logs, value and range checks, prompts and warnings if data are entered incorrectly) are built into the database to promote data quality. An audit trail is in place. Both a monitoring plan and data management plan are in place to promote data quality.

#### Confidentiality {27}

Data is collected pseudonymised using a PIN that will consist of the study site number and consecutive patient number. At each study site, there will be a PIN code list (identification log). Only the local investigators will have access to the PIN of patients treated at the study site. Study team, the safety committee supervising the study, a monitor supervising the conduct of the study and the Healthcare Inspectorate will have access to the eCRF and source file. The collection of data for medical research in the Netherlands is subject to the General Data Protection Regulation (GDPR), which replaced the Personal Data Protection Act (Wbp) in 2018, and in particular to the Medical Treatment Contracts Act. In the UK this is subject to the Data Protection Act (DPA) 2018 and the GDPR. Patient contact details (address and telephone number) will be provided to the home-care nurses, after consent by participants, by local investigators to enable distribution of study treatment and administration.

#### Plans for collection, laboratory evaluation and storage of biological specimens for genetic or molecular analysis in this trial/future use {33}

No biological specimens will be collected during this trial.

### Statistical methods

#### Statistical methods for primary and secondary outcomes {20a}

Baseline assessments, outcome and safety parameters will be summarised using simple descriptive statistics. Continuous, normally distributed variables will be expressed as means and standard deviations; continuous, non-normally distributed and ordinal variables as medians (25th–75th percentiles); and categorical variables as counts and percentages. Normality of data will be explored by a Normal Q-Q Plot and tested by the Shapiro-Wilk test. Statistical uncertainties will be quantified with two-sided 95% confidence intervals. All analyses will be based on the intention-to-treat principle, whereas the primary outcome (the number of patients in remission) and three secondary outcomes (time to improvement on disability, number of patients with improvement on disability ≥ MCID, and number of (S)AEs) will be additionally analysed on per-protocol basis. A two-sided *p* value < 0.05 will be considered statistically significant. We will not correct for multiple testing. Data analyses will be performed blinded for the allocated study intervention.

##### Primary outcome

The between-group difference in number of patients in remission (52 weeks after start of treatment) will be analysed using the chi-square test. Additionally, the primary outcome will be analysed using logistic regression, including the stratification variables (country, previous treatment) into the model. Effect size will be expressed as an adjusted odds ratio.

##### Secondary outcomes

For all secondary outcomes, except care use and health care costs, between-group differences will be assessed at both 18 and 52 weeks after start of treatment, or earlier if a preliminary endpoint is reached. Between-group differences in time to MCID-based improvement on disability are analysed by plotting Kaplan-Meier curves and comparing them using the log-rank test. Between-group difference in the number of patients with MCID-based improvement on disability (I-RODS and/or INCAT-DS) will be analysed using the chi-square test. Differences between mean change scores from baseline to follow-up with regard to disability, grip strength, muscle strength, sensory impairment, fatigue and pain will be analysed using the two-group *t* test. HRQL follow-up data (derived from the EQ-5D-5L profiles) will be assessed by repeated measurement analysis, using a linear mixed model. Additionally, the 18- and 52-week follow-up scores of these continuous outcome parameters will be analysed using multivariable linear regression models with the follow-up scores as the dependent variables, and treatment groups, the baseline values and the stratification variables as the independent variables. The repeated data structure of the secondary outcomes (data of the complete 52-week follow-up) will also be analysed using linear mixed models with treatment group membership as a fixed-effect and an appropriate random-effect structure.

##### Safety evaluation

AEs reported or assessed during study visits in the first 24 weeks (or within 6 weeks in case of a preliminary endpoint) and AEs identified on patients’ questionnaires (week 18) will be summed up and plotted separately from adverse events reported on the physicians’ questionnaires (week 52). Frequency of (S) AEs, counted once per patient, as well as description, duration, severity score, consequences for IMP administration and outcome is summarised using descriptive statistics. Frequencies will be reported as counts (percentages), per treatment group. AEs are specified by description. Frequencies of patients reporting mild and moderate AEs (combined) and severe AEs are reported. For the physician completed week 52 AE questionnaire, frequencies of patients reporting AEs and specific AEs are reported. Frequencies will be compared between the treatment groups using the chi-square test. In case of low observed counts (< 10) or expected counts (< 5), we will use the Fischer exact test instead of the chi-square test. Frequency of SAEs along with description is reported. Frequency of patients with (S) AEs leading to change in or discontinuation of treatment protocol will be reported.

##### Long-term efficacy and safety evaluation

Proportion of patients in remission, number of patients with improvement on disability equal or more than the MCID, mean change in disability and physician-reported AEs at week 104 will be analysed as described for the primary and secondary outcomes at 52 weeks. The results of the 104-week assessment will be reported separately.

#### Economic evaluation

##### General considerations

We hypothesise that a combined individualised treatment (IVIg and intravenous methylprednisolone) in CIDP patients leads to more frequent long-term remission compared to treatment with IVIg and placebo. To assess the benefits and harms of the combined individualised treatment, a prospective economic evaluation will be executed alongside the proposed randomised controlled trial. The time horizon is restricted to the initial 1-year follow-up. In addition, an analysis will be performed to extrapolate results reflecting a time horizon of 4 years, including scenarios with altered remission rates. Incremental cost-effectiveness ratios are calculated, reflecting the extra costs per additional patient in remission and the incremental costs per additional quality adjusted life year (QALY) gained. Sensitivity analyses will be performed to account for sampling variability and for differences in unit costs (95% CI, after bias corrected and accelerated non-parametric bootstrapping) [27]. Cost-effectiveness acceptability curves will be drawn to show the probability of cost-effectiveness of the individualised treatment (IVIg and corticosteroids) at various levels of willingness-to-pay per QALY up to 80,000 Euros.

##### Primary objective of the economic evaluation

The economic evaluation will be performed from a societal perspective as an incremental cost-effectiveness analysis (CEA) and an incremental cost-utility analysis (CUA) expressing the benefits of the combined treatment in terms of effectiveness (remission) and QALY’s gained. This will include an assessment of the economic impact of the implementation of the combined treatment (IVIg and corticosteroids) as replacement for IVIg treatment alone.

##### Cost-analysis

The economic evaluation includes health care costs, out-of-pocket expenses, and costs of production loss. Cost data include direct medical (e.g. drug costs (dose, frequency) of IVIg, IVMP, co-medication and co-prescription of other medication, outpatient and in-patient service costs, including (IVIg infusion related) home care), direct non-medical and indirect costs. Direct medical costs are defined as the volumes of health care resource utilisation multiplied by unit prices. Out-of-pocket expenses concerning health-related travel, informal home care, over-the-counter medication and mobility aids will be recorded. Differences in costs will be reported along with 95% CI after bias corrected and accelerated non-parametric bootstrapping [27]. All registered volumes within the participating centres will be valued according to the Dutch costing guideline for health care research [28]. Unit costs of IVIg and corticosteroids will be derived concordant with the Dutch Pharmacotherapeutic Compass and the website www.medicijnkosten.nl of the National Health Care Institute. The friction costs method will be applied to derive the costs of lost productivity, irrespective of age and sex [26, 28]. Costs will be expressed for the base year 2019 and discounting of costs (4%) and effects will be performed. Unit costs from different calendar years will be indexed with general yearly consumer price indices. Transferability of resource utilisation between the Netherlands and the UK will be weighed considering possible case-mix variations. Cost-calculations will be based on clinical pathway components related to the intervention and relapse and actual resource use for both treatments during the study period (e.g. use of IVIg and corticosteroids, in-patient visits, outpatient visits and hospital stay). Data on resources used are directly collected from the hospital information systems, clinical report forms and self-administered health and labour questionnaires (HLQ): *i*MCQ and the *i*PCQ adapted to the study population [26]. Tailoring of these questionnaires included extending the recall to cover the period until the previous completion of the questionnaire, or previous visit in case of a preliminary endpoint.

##### Patient outcome analysis

HRQL will be assessed with the EQ-5D-5L questionnaire at baseline, 6, 18, and 52 weeks or if an early endpoint is reached. The EQ-5D-5L is applied to score a patient’s health status, which is subsequently transposed in QALY’s by applying existing Dutch time trade-off based utility algorithm.

##### Missing HLQ data

When missing values concerning out-of-hospital care consultations are encountered, a value of 0 will be assumed if the questionnaire was otherwise completed. If unrealistic volumes of care are reported or assumed, we will seek to verify information by contacting patients or their care providers and/or estimated volume of care based on group averages.

#### Budget impact analysis (BIA)

##### General considerations

A BIA will be designed and executed in accordance with the International Society for Pharmacoeconomics and Outcomes Research (ISPOR) guidelines [29]. The analysis will be patient based, covering relevant health care costs observed during the 52 weeks study period. The main focus will be on the reduction of expenditures for IVIg medication related to the combined treatment. Analyses will cover relevant health care costs observed, such as continued IVIg infusions and related home care in the event of a relapse requiring treatment, during the follow-up period. Based on trial results, different remission rate scenarios will be considered including a sensitivity analysis around these confidence intervals.

##### Cost-analysis

For the BIA current unit costing guidelines for costing in health care will be applied as previously described. Budget impacts will be expressed in millions of Euros.

#### Interim analyses {21b}

Interim efficacy analyses will not be performed. An independent data and safety monitoring board (DSMB) will perform interim safety analyses, to ensure that the combination of IVIg and IVMP does not cause any undue risk for the patient. These interim analyses will be performed after enrolment of 20 and 60 patients, after and if 20 patients reach a preliminary endpoint during treatment period, in case of a mortality and at the discretion of principal/local investigator. Statistical stopping boundaries are not pre-specified. DSMB recommendations are sent to the PI of the study. If the PI partly rejects (part of) the DSMB’s recommendations, PI will provide the DSMB with a written explanation of his decision and supporting rationale within 2 weeks. If the DSMB has recommended that the study should be stopped but the PI decides to continue the study, the investigator will immediately inform the METC, the Board of Directors, the head of the department and all concerned regulatory authorities of its decision to continue the study despite the DSMB’s recommendation.

#### Methods for additional analyses (e.g. subgroup analyses) {20b}

We will not perform additional analyses.

#### Methods in analysis to handle protocol non-adherence and any statistical methods to handle missing data {20c}

In the ITT analysis, all randomised patients will be analysed in the treatment group to which they were originally allocated, irrespective of potential eligibility deviation, unblinding, non-adherence or other deviations from protocol. In the PP analysis, patients will be analysed who were included in accordance with the study protocol and who completed the treatment originally allocated. Patients in whom protocol deviations occurred will be excluded from the per protocol population, with the exception of the following deviations: patients having missed one IMP and/or IVIg administration, patients who receive their treatment out of window (up to 7 days) or patients with deviations on an infusion-administration level (i.e. partial IMP or IVIg administration (single infusion), IMP administration infused faster than the required 1 h).

##### Handling of missing data

Patients withdrawing consent after randomisation, but prior to the start of the treatment protocol, will be excluded from the analysis. In case the number of patients with missing data on the primary outcome is ≤ 1%, the primary outcome data will not be imputed. However, when the number of patients with missing values is larger than 1%, we will use multiple imputation with five imputed data sets to carry out the main analysis in the intention to treat population. We will use the imputed values for I-RODS and/or INCAT-DS to determine primary outcome if appropriate.

#### Plans to give access to the full protocol, participant-level data and statistical code {31c}

The protocol and generated datasets including statistical code are available from the corresponding author upon reasonable request.

## Oversight and monitoring

### Composition of the coordinating centre and trial steering committee {5d}

Department of Neurology of the Amsterdam UMC, location AMC, the Netherlands, is responsible for trial management as the study sponsor. UCLH are the coordinating centre for the UK.

The steering committee consists of the following persons: F. Eftimov and I.N. van Schaik from the Netherlands and M.P. Lunn and R.D.M. Hadden from the UK.

The writing committee consists of the following persons: F. Eftimov, I.N. van Schaik, S.R.M. Bus, R.J. de Haan and C.A.J.M. de Borgie from the Netherlands and M.P. Lunn, L. Zambreanu, R.D.M. Hadden, Y. Rajabally and M. Abbas from the UK.

The Independent DSMB charter are responsible for interim (unblinded) safety evaluation. A list of DSMB members is provided on page 37.

### Composition of the data monitoring committee, its role and reporting structure {21a}

Monitoring and quality assurance of the study will be performed by monitors from the CRU of the Amsterdam UMC, location AMC, in compliance with GCP. Monitoring in the UK will be set up separately according to local and national requirements. Monitoring visits include an initiation visit, before enrolment of first subject and after ethics committee/institutional review board approval, and three monitoring visits followed by a closing visit after database lock. A monitoring plan is in place to guarantee adequate and uniform study monitoring and quality assurance. Monitors are given data viewing and query rights in the eCRF. Data collected throughout the study are monitored and the working forms, including questionnaires, are checked for accuracy and completeness. Queries are generated in the eCRF when questions arose during data checks. Queries are resolved within the requested timeframe and at least before the next site visit takes place. Safety reporting, including (S) AE reports and their proper administrative handling, will be checked for accuracy and completeness.

### Adverse event reporting and harms {22}

AEs are defined as any undesirable experience occurring to a patient during the first 24 weeks of the study (treatment period + 6 weeks), whether or not considered related to study drugs (IVMP, placebo), standard care (IVIg) or co-medication (osteoporosis prophylaxis). In case study treatment is stopped before the 18-week treatment protocol is completed (i.e. in case of deterioration, switch to plasmapheresis), (S) AEs are recorded until 6 weeks after last study treatment. All AEs reported spontaneously by the patient or observed by the investigator or his staff are recorded at each follow-up visit during the treatment period. In addition, we will use structured questionnaires to assess corticosteroid and IVIg-associated AEs at 18 weeks, 52 weeks and at 104 weeks (long-term safety follow-up). In case weight gain is reported and confirmed by investigator, an increase of 3 kg or more is considered an adverse event. A SAE is any untoward medical occurrence or effect that at any dose results in death, is life threatening (at the time of the event), requires hospitalisation or prolongation of existing inpatients’ hospitalisation, results in persistent or significant disability or incapacity and is a congenital anomaly or birth defect; any other important medical event that may not result in death, be life threatening, or require hospitalisation may be considered a serious adverse experience when, based upon appropriate medical judgement, the event may jeopardise the patient or may require an intervention to prevent one of the outcomes listed above. SAEs that occur during the first 24 weeks of the study, or in the case of an early endpoint within 6 weeks of last treatment course, whether or not causally related to the treatment with IVMP must be reported immediately (within 24 h of the investigator becoming aware of the event) to the sponsor. Hospitalisation for receiving IVIg or any other CIDP treatment, such as (admittance to a hospital/day care facility for) plasmapheresis, will not be regarded a SAE. The sponsor will report the SAEs through a specific web portal (ToetsingOnline) to the accredited METC that approved the protocol, within 15 days after the sponsor has first knowledge of the SAE. SAEs that result in death or are life threatening will be reported within 7 days after the responsible investigator has first knowledge of the adverse event. Complete report is reported within 8. SAEs that occur in the UK will be also reported in the UK following national and local regulations. All AEs will be followed until they have abated, or until a stable situation has been reached. Depending on the event, follow-up may require additional tests or medical procedures as indicated, and/or referral to the general physician or a medical specialist.

### Frequency and plans for auditing trial conduct {23}

There are no plans for an audit of trial conduct. Monitoring of study management and data collection is discussed in the section ‘Interim analyses {21b}’.

### Plans for communicating important protocol amendments to relevant parties (e.g. trial participants, ethical committees) {25}

All amendments will be submitted for approval to the MREC and to the competent authority (CA). Relevant changes to protocol and/or study management are reported to study staff and, if applicable, to participants. Trial registry is updated following substantial amendments. The study sponsor will submit a summary of the progress of the trial to the accredited MREC once a year. Information will be provided on the date of inclusion of the first patient, numbers of patients included and numbers of patients that have completed the trial, SAEs/serious adverse reactions (ARs), other issues and amendments.

## Dissemination plans {31a}

Trial results will be published in a medical scientific journal. Results will be presented during (international) conferences and events organised by patient foundations.

## Discussion

The OPTIC trial is a randomised controlled trial aiming to determine whether combined induction treatment of IVIg and IVMP leads to more remissions than IVIg treatment alone. The trial was designed with IVMP as add on treatment, as most physicians and patients prefer IVIg treatment instead of first-line corticosteroids due to its rapid mode of action and side effect profile. Alternative designs, such as IVMP treatment as standard treatment and randomisation to IVIg/placebo as add on treatment, or an IVIg versus IVMP head-to head comparison, would have reduced feasibility of including sufficient patients due to expected reluctance of patients to participate. A trial directly comparing IVIg and IVMP, with a different primary outcome and inadequate power to assess differences in remission rates, has been performed previously and these long-term remission data contribute to the rationale of the current study [9, 30]. In addition, given the expected difference in time to improvement and possibility of unblinding due to corticosteroid AEs, the design of a head-to-head comparison with a primary outcome of remission would be challenging, as patients not improving in the first 1–2 months will probably drop out of the study to be treated with IVIg. This would lead to a relatively large group stopping with corticosteroids and insufficient exposure to corticosteroids to achieve remissions, as seen in the previously mentioned study [9]. Finally, a 3-arm study of IVIg, IVMP and combined IVIg and IVMP would probably provide the best evidence and guidance, but this design would have the same challenges of including sufficient patients and of early drop-out, and was not regarded feasible in this rare disease. The IVIg regimen is a shortened version of the treatment protocol in the largest IVIg trial in CIDP [13]. This IVIg treatment protocol was chosen to limit potential efficacy concerns in the control group, which may arise from different local IVIg protocols. Local protocols vary in dosing regimens, intervals and dose tapering or withdrawal strategies. It is likely that some patients are overtreated with IVIg in this 18-week treatment protocol, as some patients may only require a single loading dose or maintenance treatment at a lower dose than 1 g/kg [31]. We aim to limit potential overtreatment by stopping treatment after 18 weeks, but if the combined induction treatment proves superior, one could consider individualising treatment by shortening the IVIg treatment and/or reduce the dose regimens depending on treatment response. The primary outcome, remission at 52 weeks, is measured using the I-RODS and the adjusted INCAT-DS. We chose to include both outcome measures to assess disability as relevant changes may be missed in patients if only one of these is used, as was shown in both the IVIg withdrawal as well as subsequent restabilization phase of the PATH study, which used a combination of both outcome measures to assess relevant changes [32, 33]. Combining two outcome measures and using a(n individual) MCID-based approach, we hope to limit potential delay when treatment is required in the event of a relapse. Precautions have been taken to limit potential side effects and unblinding, such as excluding significant cardiovascular co-morbidity and diabetes from enrolment. Potential synergistic effects of IVIg and IVMP regarding (S) AEs have not been previously mentioned in CIDP studies. A study comparing the addition of IVMP/placebo to IVIg in Guillain-Barré syndrome (GBS) did not report significant differences in (S) AEs between the two treatment arms [34]. All patients in the Netherlands will be treated with co-medication to reduce the chance of corticosteroid-induced osteoporosis. In the UK, only patients at risk will be offered co-medication (regardless of subsequent treatment allocation), following national guidelines. This is based on the fracture risk assessment (FRAX score) and counselling regarding the side effects of alendronate acid. We excluded several patient categories for safety reasons (risk of thrombosis) and high risk of unblinding (diabetes). If the combination treatment proves superior, this treatment could be considered in patients that are currently excluded, if AEs due to IVIg and/or corticosteroids are considered manageable. The OPTIC trial started enrolling participants in February 2018. Due to protracted legal negotiations and institutional review board (IRB)/MREC approval, study start was delayed in some Dutch centres as well as in the UK. In the UK, study was approved by the MHRA (Medicines and Healthcare products Regulatory Agency UK) on 29 March 2019, the South Central – Oxford REC 10 May 2019 and by the Health Research Authority (HRA) and Health and Care Research Wales (HCRW) 16 May 2019. This has resulted in a slower rate of inclusion than anticipated. Overall, most eligible patients screened for the study who meet the inclusion criteria are willing to participate in the study. Screen failures are predominantly due to the presence of diabetes, which is an exclusion criterion, or previously administered treatment. As this trial focuses on patients with active disease, instructing non-participating and referring hospitals not to start treatment for CIDP is probably one of the biggest challenges to ensure a steady rate of inclusions. Regarding trial oversight, monitoring of visits so far did not report any operational issues regarding data quality and collection or protocol adherence. The DSMB, after an SAE and after unblinded review of safety data following the first 20 inclusions, did not advise any changes to study management or safety measures. During the course of the study, five amendments were made to the protocol and overall conduct of the study. The first amendment (approved 25 May 2018) added a ‘known pulmonary embolism or other deep venous thrombosis in patient’s medical history, without current anticoagulant therapy’ to the exclusion criteria after a SAE in the first patient enrolled in the study. The DSMB charter was modified to allow for ad hoc meetings at the request of PI. The patient information sheet was changed to include specific warning signs for (deep venous or arterial) thrombosis. The protocol was changed to allow local pharmacies to source IVMP and co-medication. The second amendment (2 July 2018) implemented a change of PI in the Radboud University Medical Centre. The third amendment (15 February 2019) included a cap on the total amount of IVIg administered (standard care) of 200 g, reflecting local practices in the UK. A range of appropriate dosing was added to reflect local practices regarding co-medication dosing the UK. A window of 2 days (earlier and later) was added in the planning of home care treatment scheduling. An emergency unblinding procedure was added. A 2- and 4-week window was added for the week 52 and week 104 study visits. Language was added to specify that hospital (or day care) admittance for further CIDP treatment (in case of a relapse, deterioration during treatment protocol) would not be considered a SAE. The fourth amendment (5 August 2019) further specified the text of two exclusion criteria (4 and 11) and added a further two (5 and 6) during the process of ethical approval in the UK. These additions were not made after a SAE or other safety concerns. In the fifth amendment (15 May 2020), changes to protocol were made to ensure safe study assessments in light of the COVID-19 outbreak. Enrolment of patients was temporarily stopped. Patients in the treatment phase of the trial were allowed to continued treatment. Study visits were allowed to be conducted as a telephone consultation, with questionnaires sent by mail or through Castor EDC. Week 18 and 52 visits were conducted by phone, in clinic or as home visits (the latter two if safety permitting). The amendment included a provision which allowed for a return to normal protocol when restrictive measures were (gradually) lifted. Screening logs were maintained during the temporary enrolment stop.

## Trial status

The first participant was enrolled 19 February 2018. There are currently 34 patients enrolled, out of 96, and estimated completion of recruitment is end of 2022. The current version of the protocol is 3.5, dated 15 March 2020.

## Abbreviations

AE: Adverse event
AMR: AMC Medical Research BV
AR: Adverse reaction
BIA: Budget impact analysis
CA: Competent authority
CEA: Cost-effectiveness analysis
CIDP: Chronic inflammatory demyelinating polyradiculoneuropathy
CRU: Clinical Research Unit
eCRF: (electronic) Case report form or working document
CUA: Cost-utility analysis
DPA: Data Protection Act
DSMB: Data Safety Monitoring Board
EFNS/PNS: European Federation of Neurological Societies/Peripheral Nerve Society
FRAX: Fracture risk assessment
GBD: Guillain-Barré syndrome
GCP: Good Clinical Practice
GDP: Good Distribution Practice
GDPR: General Data Protection Regulation
GMP: Good Manufacturing Practice
HCRW: Health and Care Research Wales
HRA: Health Research Authority
HRQL: Health-related quality of life
IC: Informed consent
iMCQ: iMTA Medical Consumption Questionnaire
IMP: Investigational Medicinal Product
INCAT-DS: Inflammatory Neuropathy Cause and Treatment Disability Scale
INCAT-SS: Inflammatory Neuropathy Cause and Treatment Sensory sum score
iPCQ: iMTA Productivity Cost Questionnaire
I-RODS: Inflammatory Rasch-Overall Disability Scale
ISPOR: International Society for Pharmacoeconomics and Outcomes Research
IU/IE: International Unit
IVIg: Intravenous immunoglobulins
IVMP: Intravenous methylprednisolone
MCID: Minimal Clinical Important Difference
METC: Medical Research Ethics Committee (MREC)
MHRA: Medicines and Healthcare products Regulatory Agency UK
MRC: Medical Research Council
NHS: National Health Services
PI: Principal investigator
PIN: Personal identification number
PI-NRS: Pain intensity numeric rating scale
PPI: Proton-pump inhibitors
QALY: Quality-adjusted life years
R-FSS: Rasch-built Fatigue Severity Scale
(S)AE: (Serious) adverse event
SmPC: Summary of Product Characteristics
SOP: Standard Operating Procedure
Sponsor: Party that commissions the organisation or performance of the research, for example an academic hospital, scientific organisation or investigator, a party that provides funding for a study but does not commission it is not regarded as the sponsor, but referred to as a subsidising party
SUSAR: Suspected Unexpected Serious Adverse Reaction
UCLH: University College London Hospitals
UK: United Kingdom
UF: Unblinding form
URF: Unblinding Request Form
Wbp: Personal Data Protection Act
WMO: Medical Research Involving Human Subjects Act
ZonMW: Netherlands Organization for Health Research and Development for health related funding
